# Frequency-domain broadband near-infrared spectroscopy for noninvasive monitoring of fluid volume status during hemodialysis

**DOI:** 10.1117/1.BIOS.3.1.015003

**Published:** 2026-02-04

**Authors:** Diana Suciu, Thao Pham, Lina Lin Wei, Isaac Hoekstra, Pranav Yadati, Vipul C. Chitalia, Darren Roblyer

**Affiliations:** a Boston University, Department of Biomedical Engineering, Boston, Massachusetts, United States; b Boston University Medical School, Boston Medical Center, Department of Medicine, Renal Section, Boston, Massachusetts, United States; c Boston Veterans Affairs Healthcare System, Boston, Massachusetts, United States; d Boston University, Department of Electrical and Computer Engineering, Boston, Massachusetts, United States

**Keywords:** near infrared spectroscopy, absorption, scattering, tissues, end-stage kidney disease, nephrology, hemodialysis, water, wearable, diagnostic, spectroscopy, diffuse optics

## Abstract

**Significance:**

Poor fluid management during hemodialysis in patients with end-stage kidney disease contributes to adverse clinical outcomes. Current tools for assessing volume status are limited, and there is a need for objective measurement techniques.

**Aim:**

We aim to evaluate the ability of a hybrid frequency-domain (FD) and broadband continuous-wave near-infrared spectroscopy (FD-Bb-NIRS) system to monitor tissue changes during hemodialysis and identify markers associated with intradialytic adverse events.

**Approach:**

A custom-built FD-Bb-NIRS system was used to acquire continuous optical measurements. The system utilized FD laser diodes (730, 785, 852, and 940 nm) and broadband illumination (700 to 1000 nm). These measurements were integrated using model-based analysis to provide broadband absorption and reduced scattering spectra. Beer’s law was used to extract tissue chromophore concentrations.

**Results:**

Across 27 subjects, the water ratio ([water]/([water] + [lipid])) showed significant differences between subjects with and without intradialytic adverse events (p=0.0331), with a decrease observed in patients without adverse events. In addition, tissue scattering parameters contributed to the multivariate classification of adverse event discrimination.

**Conclusions:**

The FD-Bb-NIRS device successfully measured changes in tissue fluid dynamics during hemodialysis. Longitudinal water ratio measurements show promise as optical markers for identifying patients at risk of adverse events.

Statement of Discovery This work utilized combined frequency-domain and broadband continuous-wave near-infrared spectroscopy to monitor tissue optical properties in patients undergoing hemodialysis. We discovered that tissue water changes were significantly different between patients who experienced intradialytic adverse events and those who did not. These findings support the potential for a noninvasive optical metric for improving patient monitoring and could enable the development of early prediction modalities for adverse events during hemodialysis.

## 1 Introduction

Chronic kidney disease (CKD) affects approximately one in seven adults in the United States, representing an estimated 30 million individuals.^1^ Of these, over 700,000 are classified as end-stage kidney disease (ESKD) patients.^1^^,^^2^ In ESKD, renal function becomes insufficient to sustain life without intervention.^1^^,^^2^ Kidney transplantation remains the optimal long-term treatment for ESKD; however, due to organ shortages and transplant waitlists exceeding 5 years, ESKD patients rely on dialysis as their primary lifeline.^1^^,^^2^ Hemodialysis, the most common renal replacement therapy, removes excess solutes, toxins, and water (also called volume) from the bloodstream. In 2017 and 2018, 70% of the over 700,000 Americans with CKD were on hemodialysis.^1^^,^^3^ Patients with ESKD accumulate fluid in between hemodialysis sessions; its removal is a quintessential function of hemodialysis. Achieving a clinically optimal fluid balance during hemodialysis remains one of the most challenging aspects of clinical ESKD management. The median survival for patients undergoing maintenance hemodialysis is ∼5 to 7 years, with a 5-year survival rate of ∼50%.^4^^,^^5^ A major contributor to mortality in patients with CKD is cardiovascular complications driven by inadequate volume management during hemodialysis.^6^^,^^7^ Even mild fluid overload is an independent risk factor for all-cause and cardiovascular mortality in CKD patients. Excess fluid removal results in hypotension, muscle cramps. These clinical complications result in a premature termination of the hemodialysis session in close to 20% patients, compromising patients’ long-term vitality.^8^ Nearly half of all hemodialysis patient deaths and a quarter of all hospitalizations are associated with cardiovascular disease.^7^^,^^9^ With an estimated 20% to 50% of all ESKD patients undergoing inaccurate fluid removal during treatment,^10^ there is a critical need for improved, long-term, sustainable support strategies for this patient population.

A fundamental challenge in hemodialysis care is the accurate assessment of a patient’s “dry weight,” or the weight at which a patient is euvolemic, meaning that they have neither too much nor too little volume.^11^^,^^12^ The current standard-of-care techniques for volume assessment, or monitoring when a patient achieves “dry weight,” are often based on subjective symptoms such as cramping, dizziness, or nausea and clinical signs such as edema and inter-dialysis weight changes, which can occur when fluid is removed too quickly or in too high a quantity during treatment.^11^ This approach is imprecise and is subject to change based on the status of the patient’s health. Prior studies have shown that poor volume assessment results in erroneous ultrafiltration, causing up to 50% of patients to develop dialysis-related hypotension and experience a poor quality of life after treatment.^10^^,^^13^^,^^14^ Despite the recognized importance of accurate volume assessment, current methods remain subjective, crude, and generally unreliable in routine clinical practice.^12^^,^^15^^–^^17^ Volume assessment during hemodialysis is a research priority by the Kidney Health Initiative, given that there are currently no quantitative standards for monitoring the volume status of patients undergoing treatment.^18^^,^^19^

Near-infrared spectroscopy (NIRS) offers a potential solution to the unmet clinical need of noninvasive, objective fluid volume monitoring during hemodialysis. NIRS uses light propagation in highly scattering media to measure tissue optical properties in the near-infrared (NIR, 650 to 1000 nm) wavelength range. NIRS has been widely applied across biomedical fields, including oncology, neuroscience, and clinical diagnostic care.^20^ Four main NIRS modalities are used in biological measurements: continuous-wave (CW), temporal frequency-domain (FD), spatial frequency domain (SFDI), and time-domain (TD).^20^^–^^24^ Of these, only temporal (FD-NIRS and TD-NIRS) and spatial (SFDI) modalities allow for the independent quantification of absolute tissue absorption ($\mu_{a}$) and reduced scattering ($\mu_{s}^{\prime }$) properties, with FD-NIRS accomplishing this by measuring the amplitude and phase shift of intensity-modulated light after it travels through tissue.^20^^,^^25^^,^^26^ Although CW-NIRS systems are simpler, more compact, and capable of covering a broad spectral range, they cannot independently resolve reduced scattering and absorption. Therefore, CW systems are typically limited to monitoring relative trends in absorption or chromophore concentration.^27^^–^^29^ To overcome the trade-off between quantitative accuracy and broad spectral range, hybrid FD + CW systems have been developed, enabling the recovery of absolute broadband $\mu_{a}$ and $\mu_{s}^{\prime }$ spectra.^30^^,^^31^ Prior hybrid FD + CW NIRS techniques, namely, FD-Bb-NIRS, have largely focused on quantifying concentrations of oxygenated and deoxygenated hemoglobin,^21^^,^^32^^–^^36^ but there has been a growing interest in using diffuse optical techniques to measure tissue concentrations of water and lipids.^37^^–^^40^

In this study, we present the findings from a clinical study employing a custom-built frequency-domain broadband near-infrared spectroscopy (FD-Bb-NIRS) system for real-time monitoring of tissue physiology during hemodialysis. To our knowledge, no previous studies have utilized a combined FD + CW NIRS system for continuous monitoring of tissue optical properties during hemodialysis nor have they evaluated such measurements in the context of categorizing adverse clinical outcomes. Our findings demonstrate that tissue-level changes in relative tissue water fraction and absolute reduced scattering parameters exhibit statistically significant associations with hemodynamic instability, outperforming the clinically available tool Crit-Line (Fresenius Medical Care, Bad Homburg, Germany) in identifying dialysis sessions in which adverse events occurred. These results suggest the potential for noninvasive optical techniques to enhance fluid status assessment and improve patient safety in reducing adverse events and treatment efficacy through adequate volume removal in the hemodialysis patient population.

## 2 Materials and Methods

### 2.1 Instrumentation

**Fig. 1:**
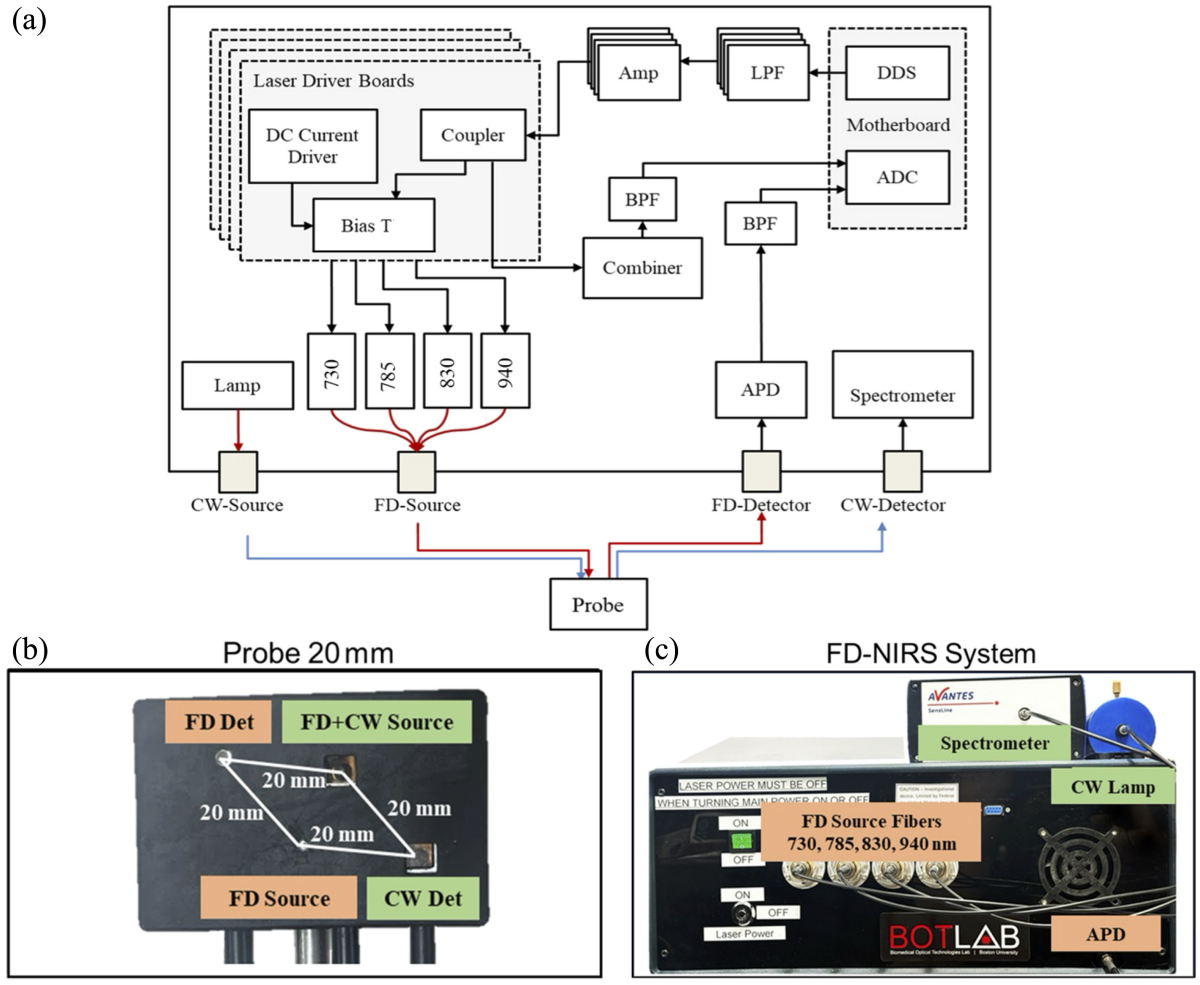
(a) Schematic diagram of the custom FD-Bb-NIRS system. Key components include the following: ADC, analog-to-digital converter; APD, avalanche photodiode; DC, direct current; DDS, direct digital synthesizer; LPF, low-pass filter; Amp, amplifier; BPF, bandpass filter. Frequency-domain (FD) sources (730, 785, 930, and 940 nm) and FD detector are indicated by red lines, and continuous-wave (CW) source and detector are indicated by blue lines. (b) Probe containing the FD and CW source and detector fibers with a source-detector separation (SDS) of 20 mm. (c) FD-Bb-NIRS clinical system. The black enclosure houses the electronics controlled via a Windows 10 laptop.

The clinical measurement system FD-Bb-NIRS combined FD and broadband NIRS components [Fig. 1(a)]. Details of the custom digital FD-NIRS component have been previously described.^41^ Briefly, the FD component of the system used illumination wavelengths of 730, 785, 830, and 940 nm (LP730-SF15, LP785-SF20, LP830-SF30, LP940-SF30, Thorlabs, Newton, New Jersey, United States). The FD laser illumination power ranged from 9.5 to 14 mW per laser, depending on the wavelength, such that the system’s combined FD lasers power output was within the American National Standards Institute (ANSI) safety standard. Direct digital synthesizer (DDS) boards were used to simultaneously modulate each laser diode at the selected discrete frequencies between 119 and 134 MHz. A broad-spectrum tungsten halogen lamp (HL-2000-HP-B, Ocean Optics, wavelength range from 360 to 2400 nm) was used as the illumination source for the CW measurements.

Custom optical fibers were made by Fiberoptic Systems Inc. (Simi Valley, California, United States) for both FD and CW measurements. All fibers terminated at a right angle so they could lie flat against the subject’s skin. Two source and detector fibers were used to increase optical power to allow for a 20 mm source detector separation (SDS), allowing for sensitivity to tissue depths of at least several millimeters.^38^^,^^42^ A custom fiber holder was 3D printed to hold the fibers for clinical measurements, as seen in Fig. 1(b). The source FD fibers were 400  μm diameter fibers with a numeric aperture (NA) of 0.55 to deliver light to the tissue. A 2.3 mm fiber (NA: 0.55) was used for FD detection, and a 1 mm fiber (NA: 0.55) was used for CW source and detection. For FD-NIRS detection, a 3 mm diameter silicon avalanche photodiode (APD) (S11519, Hamamatsu, Hamamatsu, Japan) was used to detect laser light. A 250 mega-sample per second analog-to-digital converter (ADC) was used to digitize the APD signal. The Goertzel algorithm was implemented in the system’s microprocessor to calculate the phase and amplitude of the FD reflectance measurements at each wavelength. The CW-NIRS emitted broadband light was detected using a 2048-pixel CCD spectrometer (AvaSpec-HS2048XL, Avantes, Apeldoorn, the Netherlands). The grating and slit width were selected to achieve a usable measurement range of 700 to 1000 nm with a spectral resolution of 7.5 nm full width at half maximum. A calibration procedure was used to remove the instrument response function of the clinical system as previously described using the solid silicone phantom for FD calibration and a Spectralon standard (Labsphere, North Sutton, New Hampshire, United States) for CW calibration.^43^ The clinical system was controlled by a custom interface written in MATLAB (MathWorks Inc., Natick, Massachusetts, United States) and operated on a Windows 10 laptop. The FD and CW data were collected sequentially as described in our previous work,^43^ at a final sampling rate of 110 to 1200 ms per measurement (subject dependent). The assembled system could fit on a portable cart that was brought bedside during clinical measurements [Fig. 1(c)].

### 2.2 Clinical Data Acquisition

A clinical study was conducted to assess the ability of the FD-Bb-NIRS system in monitoring tissue changes during hemodialysis and to identify potential physiological markers associated with intradialytic adverse events. Participants for this study were recruited from the inpatient hemodialysis care center patient population at Boston Medical Center from July 2023 to November 2024. All measurements were conducted under an institutionally approved protocol (BU BMC IRB H-41696). The inclusion criteria encompassed individuals above the age of 18 of any race, gender, or ethnicity with ESKD who were prescribed fluid removal during their inpatient hemodialysis treatment. The exclusion criteria included anyone with below-knee amputations, who had restless leg syndrome or medical anxiety that included symptoms of restlessness. In addition, subjects who only had blood cleaning with no fluid volume removed were excluded from the study. All participants consented to the study in person. In total, 27 participants consented to the study (Table 1 shows further participant demographic information). Every session was considered independent, and no subjects underwent more than one session.

**Table 1:** Demographic and clinical characteristics of study participants (N=27). Values are presented as mean ± standard deviation unless otherwise indicated. Fluid removed represents the average ultrafiltration volume per session. Crit-Line refers to subjects whose hematocrit was continuously monitored during dialysis via the Fresenius Crit-Line IV Monitor.

	Patients	
Total	N=27	
Crit-line	N=19	
Sex		
Male	N=16	
Female	N=11	
Race		
Black	N=19	
White	N=4	
Other	N=4	
Ethnicity		
Hispanic	N=7	
Non-Hispanic	N=19	
Other	N=1	
	Mean ± Stdev	
Age	58.8 ± 14.2 (Y.O.)	
Height	1.7 ± 0.1 (m)	
Weight	78.9 ± 15.4 (kg)	
Fluid removed	1.6 ± 0.8 (L)	
	Median	Range
Dialysis duration	3	1 to 4 (h)

**Fig. 2:**
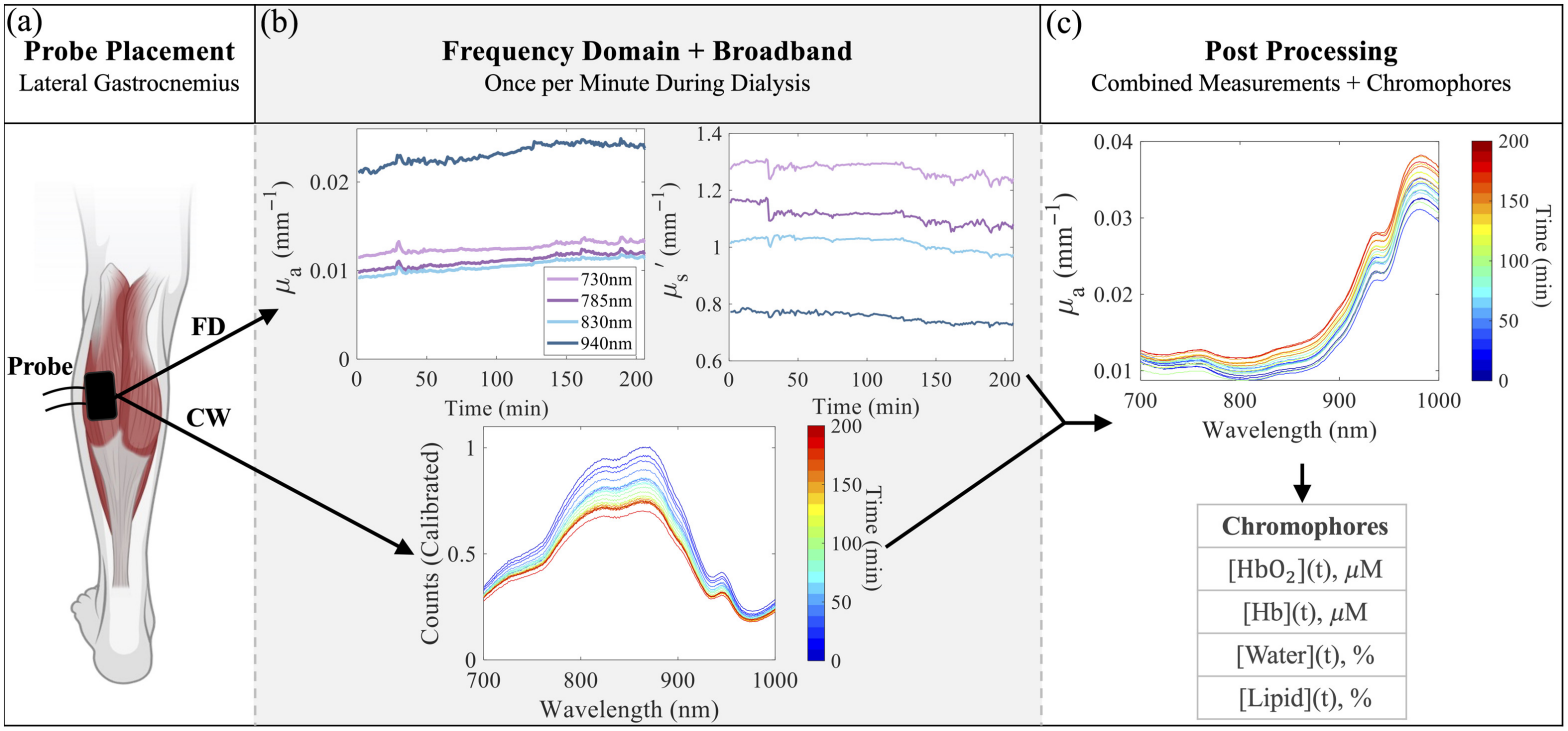
Illustration of the experimental workflow for acquiring and processing optical measurements during hemodialysis using the FD-CW-NIRS clinical system. (a) The probe was positioned over the main muscle body of the lateral gastrocnemius muscle. (c) FD and CW measurements were acquired once per minute throughout the dialysis session. The top panels show representative time traces of FD absorption ($\mu_{a}$) and reduced scattering ($\mu_{s}^{\prime }$) at 730, 785, 830, and 940 nm. The bottom panel displays a representative calibrated CW reflectance spectra at wavelengths from 700 to 1000 nm recorded over time. (c) Broadband absorption spectra ($\mu_{a}$) from 700 to 1000 nm were reconstructed from the calibrated broadband reflectance spectra using MC-LUT, enabling chromophore quantification. Chromophore concentrations were estimated using Beer’s law. Created in part using BioRender.^44^

The measurement setup is shown in Fig. 2. For each subject, their lateral gastrocnemius muscle was palpated to identify the main head of the muscle body. The probe containing the source and detector fibers was placed on the skin above the muscle body, oriented longitudinally to the muscle fibers, and secured using Tegaderm tape. This location was chosen based on previous works by Colucci et al., where it was observed with MRI imaging of the lower extremities that changes in the lateral gastrocnemius corresponded well to overall fluid status during hemodialysis.^45^ Subjects were reclined in a hospital bed throughout the duration of dialysis. FD and CW measurements were taken sequentially once a minute over the entire duration of dialysis. During this time, intradialytic adverse events, clinical interventions, and visible movements or postural changes were timestamped and logged for post-processing data discrimination and motion correction. In addition, blood pressure (BP), heart rate, and ultrafiltration rate were recorded every 15 min.

Intradialytic adverse events were defined as follows: vomiting, cramping, dizziness, headache, shortness of breath, sweating, stroke, fluid return, or intradialytic hypotension (IDH). Subjective adverse event experiences such as nausea, dizziness, and cramping were classified as adverse events by our clinical collaborators prior to the commencement of the clinical trial, as well as existing literature.^11^^,^^12^^,^^15^^–^^17^^,^^46^ Documentation of these events came from verbal reports from subjects, which were noted and timestamped by trial administrators. IDH is defined by the National Kidney Foundation as a decrease in systolic blood pressure (SBP) by ≥20  mmHg or a decrease in mean arterial pressure (MAP) by 10 mm Hg associated with additional symptoms described in the National Kidney Foundation’s Kidney Disease Outcomes Quality Initiative (NKF KDOQI) guidelines.^46^

### 2.3 Data Processing

The post-processing pipeline for the clinical system is adapted from a previously described algorithm.^43^ In summary, the data processing pipeline for the FD-CW-NIRS system utilized a Monte Carlo-based look-up table (MC-LUT) approach^42^^,^^43^^,^^47^ for a homogeneous medium with semi-infinite geometry to integrate FD and CW NIRS to recover tissue broadband absorption coefficient ($\mu_{a}$) and reduced scattering coefficient ($\mu_{s}^{\prime }$) over the 700 to 1000 nm spectral range. The power law [Eq. (1)] is the empirical description of the wavelength dependence of scattering in tissue and yields the scattering amplitude (a) and scattering power (b), where λ is the desired wavelength of $\mu_{s}^{\prime }$, and $\lambda_{0}$ is the reference wavelength (800 nm).

$$\mu_{s}^{\prime }=a(\frac{\lambda }{\lambda_{0}}{)}^{-b}.$$ (1)

Tissue chromophore concentrations ($\left[{\mathrm{HbO}}_{2}\right]$, [Hb], [water], [lipid]) were determined by least-squares fitting of the full $\mu_{a}$ spectrum using Beer’s law. The fitting method for chromophore concentrations was carried out with an assumption of a spectrally constant background absorption. The fit assumed that the overall absorption spectrum was modeled as the sum of absorption contributions from $\left[{\mathrm{HbO}}_{2}\right]$, [Hb], [water], [lipid], [melanin], the chromophore extinction coefficients (ε), and the constant background absorption, as shown below

$$\mu_{a\text{Tissue}}=\varepsilon_{\mathrm{HbO}}[\mathrm{HbO}]+\varepsilon_{\mathrm{Hb}}[\mathrm{Hb}]+\varepsilon_{\text{Water}}[\text{Water}]+\varepsilon_{\text{Lip}}[\text{Lipid}]+\varepsilon_{\mathrm{Mel}}[\text{Melanin}]+\mu_{a\text{Background}}.$$ (2)

Melanin concentration was calculated at baseline (pre-dialysis) and then was assumed to remain constant over the course of treatment. The chromophore concentrations and background absorption were then calculated over time using the assumed constant [melanin] value. Figure S1 in Supplementary Material 1 shows the measured [melanin] at baseline across self-reported race categories. The constant background absorption was added to account for tissue heterogeneity or missing chromophores.^48^ Extinction coefficients of the extracted chromophores are based on values from the literature.^37^^,^^49^^–^^52^

**Fig. 3:**
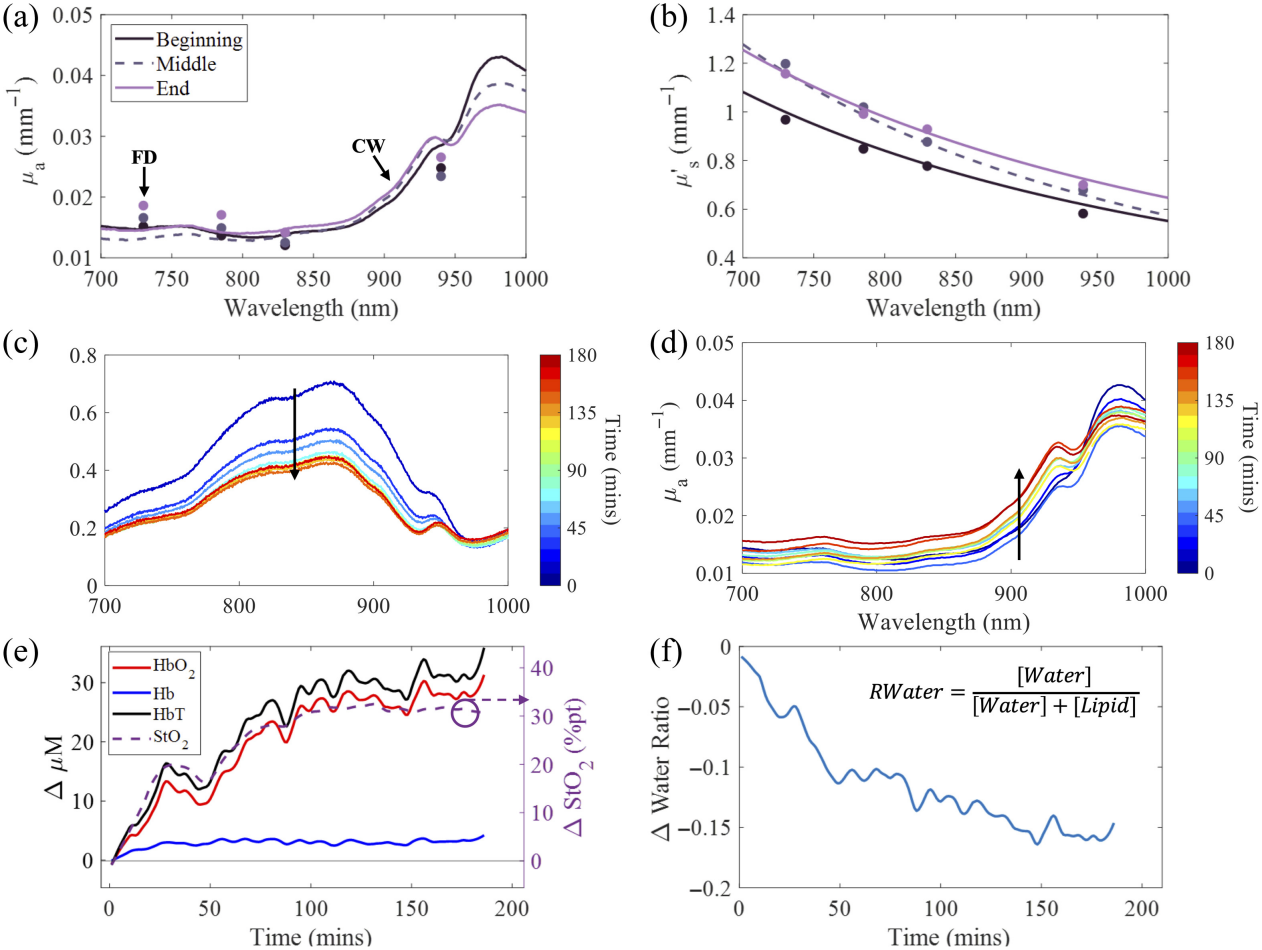
Example data from one subject, including their extracted (a) absorption coefficient spectra ($\mu_{a}$) and (b) reduced scattering coefficients ($\mu_{s}^{\prime }$) at three time points (0, 90, and 180 min). Discrete frequency-domain (FD) wavelengths (730, 785, 830, and 940 nm) are shown as data points, whereas continuous-wave (CW) data span the full 700 to 1000 nm range. (c) Calibrated CW reflectance spectra over time and (d) Broadband $\mu_{a}$ spectra, color-coded from blue (start of dialysis) to red (end), arrows show the dominant trend. (e) Temporal display of the relative chromophore concentrations changes over hemodialysis: oxyhemoglobin ($\left[{\mathrm{HbO}}_{2}\right]$), deoxyhemoglobin ([Hb]), total hemoglobin (HbT), and tissue oxygen saturation (${\mathrm{StO}}_{2}$). (f) Change in tissue water ratio (RWater).

A transient artifact reduction algorithm (TARA) algorithm was applied to the extracted chromophore measurements to mitigate motion-induced artifacts in our longitudinal tissue measurements.^53^ The motion artifacts identified by the TARA algorithm corresponded closely with the timestamped instances of observed motion (described in Sec. 2.2). For Figs. 3(e) and 3(f), local regression smoothing was applied using weighted linear least squares and a second deg polynomial model, implemented with a moving window model that spans 5% of the data set at a time.

## 3 Results

### 3.1 Subject Overview

A total of 27 participants were enrolled in the study (Table 1). The cohort included 16 male and 11 female subjects. The racial distribution was predominantly Black (N=19), with smaller representations from White (N=4) and other racial groups (N=4). The mean age of participants was 58.8±14.2 years, with an average height of 1.7±0.1  m and a mean body weight of 78.9±15.4  kg. This demographic and clinical profile reflects a representative inpatient population receiving hemodialysis at the Boston Medical Center.

An example dataset from a single subject who experienced no adverse events, illustrating the post-processing results and physiological trends during dialysis, is shown in Fig. 3. Figures 3(a) and 3(b) show absolute FD and broadband $\mu_{a}$ and $\mu_{s}^{\prime }$ spectra at three discrete time points (0, 90, and 180 min) during hemodialysis. There is an overall increase in $\mu_{a}$ and a decrease in $\mu_{s}^{\prime }$. The increase in absorption, further emphasized in Fig. 3(d), correlates with the increase in $\left[{\mathrm{HbO}}_{2}\right]$ observed in Fig. 3(e). This subject exhibited a progressive increase in tissue oxygen saturation (${\mathrm{StO}}_{2}$) and oxyhemoglobin concentration $\left[{\mathrm{HbO}}_{2}\right]$ over the entire course of hemodialysis. These trends are consistent with previously observed physiological responses during hemodialysis.^32^^,^^54^

In addition to changes in tissue oxygenation, changes in tissue water concentration were considered. The water ratio, as defined by Lam et al.,^38^ [water] / ([water] + [lipid]), was used here [Fig. 3(f)]. We have found that this metric provides a temporally stable and less noisy measurement as compared with direct [water] measurements. In this subject, the water ratio showed a net decrease over the dialysis session, potentially reflecting fluid redistribution during effective ultrafiltration. Data outside six mean absolute deviations were assigned a weight of zero. The temporal trends in optical properties and chromophore concentrations observed in this subject were broadly representative of cohort-wide responses, capturing the general trends in increasing absorption, decreasing reduced scattering, and progressive changes in $\left[{\mathrm{HbO}}_{2}\right]$, ${\mathrm{StO}}_{2}$, and tissue water content during dialysis. The difference in extracted chromophore values between the beginning and end of dialysis for all subjects is shown in Fig. S2 in Supplementary Material 1.

### 3.2 Chromophore Concentrations During Adverse Versus Nonadverse Events

Subjects were stratified into two subgroups: those who experienced an adverse event during treatment (N=18), as defined in Sec. 2.2, and those who completed dialysis without an adverse event (N=9). Demographic and treatment characteristics for each group are summarized in Table S1 and Fig. S3 in Supplementary Material 1. No statistically significant differences were observed between groups in terms of ultrafiltration volume or other baseline characteristics.

**Fig. 4:**
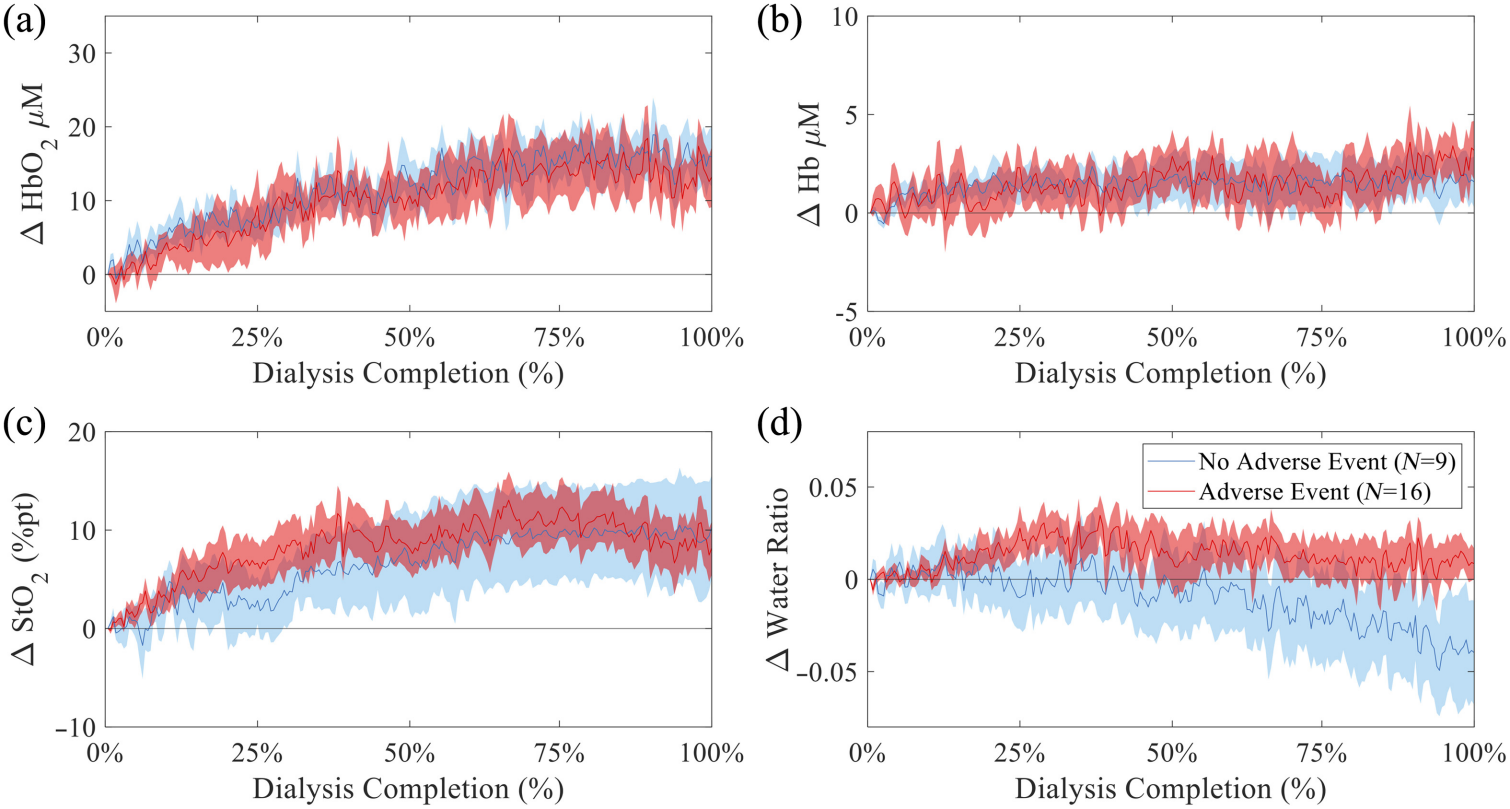
Time-normalized traces (mean ± standard error) comparing chromophore extractions of subjects with adverse events (red, N=16) and without (blue, N=9) (a) Δ $\left[{\mathrm{HbO}}_{2}\right]$, (b) Δ [Hb], (c) Δ ${\mathrm{StO}}_{2}$, (d) Δ water ratio. Two subjects with short-duration sessions (t≤114  min) were excluded.

**Fig. 5:**
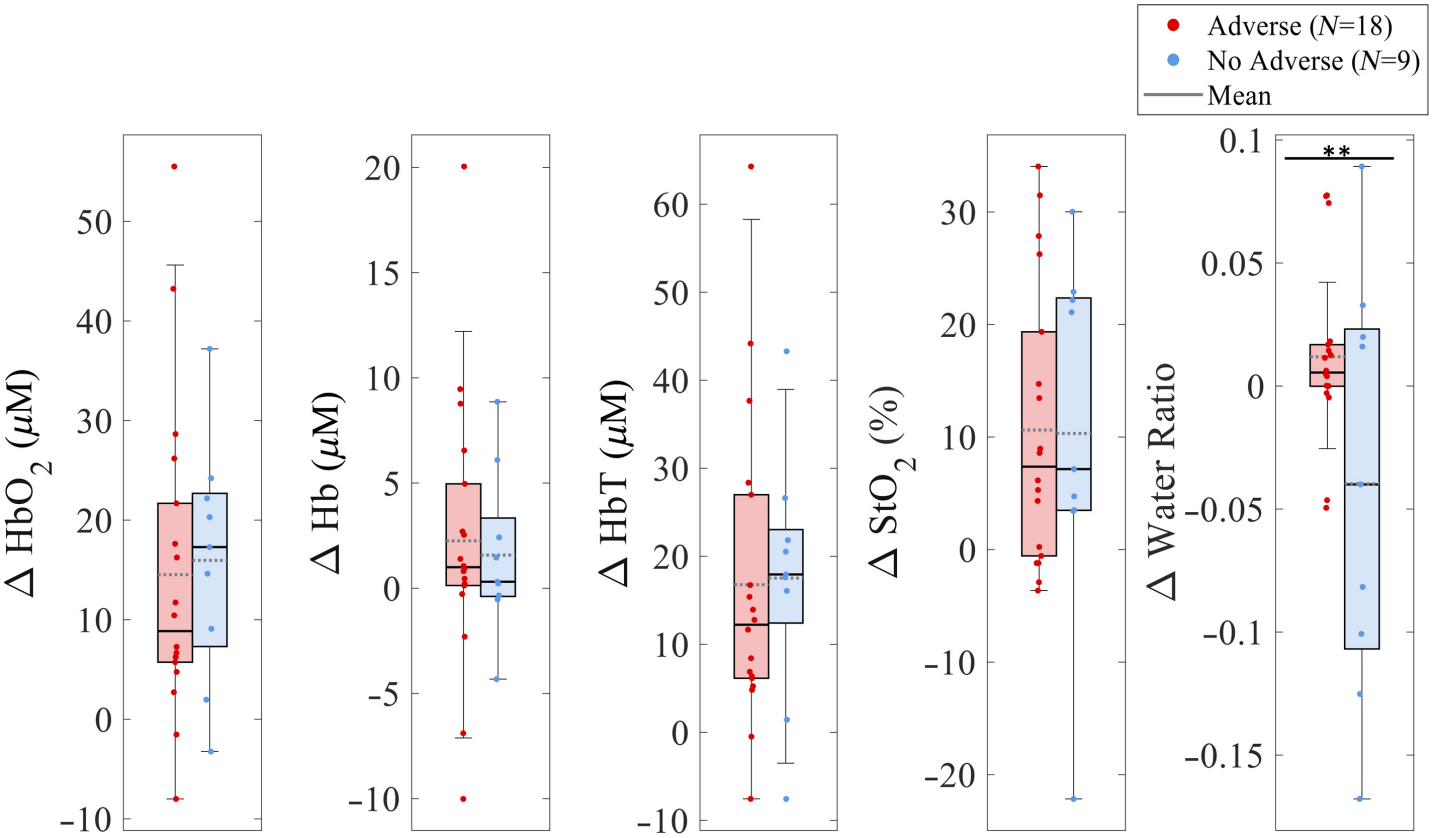
Boxplots of subject values and group means for the absolute Δ $\left[{\mathrm{HbO}}_{2}\right]$, Δ [Hb], Δ [HbT], Δ ${\mathrm{StO}}_{2}$, and Δ water ratio from the beginning to the end of dialysis. Adverse event subjects are shown in red (N=18), and nonadverse event subjects in blue (N=9). A significant difference was observed for Δ water ratio (p<0.05). No other parameters reached statistical significance.

Group-averaged dynamics of major chromophores and tissue water ratio were then analyzed over the normalized time course of hemodialysis to identify trends associated with adverse events. These longitudinal trends are presented in Fig. 4, which displays group means ± standard error for all subjects over normalized time (% dialysis completion). The longest dialysis duration was 227 min; two subjects with treatment durations less than half (t≤114) this time were excluded from the longitudinal analysis to ensure sufficient temporal resolution for trend comparisons. Across hemodialysis, both groups demonstrated a general increase in the $\left[{\mathrm{HbO}}_{2}\right]$ with respect to baseline (i.e., $\Delta [{\mathrm{HbO}}_{2}]$) [Fig. 4(a)], with slightly higher variability in the adverse events group, though overall both groups had a similar total mean change for all subjects, with 14.52±15.82  μM for subjects with adverse events and 15.96±12.21  μM (Fig. 5) for subjects without adverse events. Δ[Hb] levels showed slightly divergent patterns [Fig. 4(b)], remaining near baseline or slightly negative for subjects without adverse events, whereas subjects with adverse events had a higher increase over time. Δ[Hb] increased by an average value of 2.25±6.49  μM for subjects with adverse events and 1.57±3.88  μM for those without adverse events during dialysis (Fig. 5). Changes in Δ ${\mathrm{StO}}_{2}$ [Fig. 4(c)] exhibited a more rapid rise within the first 25% of dialysis in subjects who experienced adverse events; however, by the end of treatment, both groups reach a comparable absolute change in ${\mathrm{StO}}_{2}$ at an average of 10.62±12.41% pt for all subjects in the adverse events groups and 10.32±15.78% pt for the nonadverse group (Fig. 5).

Most notably, the Δ water ratio [Fig. 4(d)] diverged substantially between groups. The group without adverse events exhibited a gradual decline, with an average of −0.037±0.085, whereas the adverse event group shows an increase of 0.012±0.035 (Fig. 5). A summary table of the Δ chromophores means, standard deviations, and statistics (2-sided t-test) is provided in Table S2 in Supplementary Material 1. The time courses of Δ [water] and Δ [lipid] extractions used to calculate Δ water ratio are presented in Fig. S4 in Supplementary Material 1.

In addition to assessing temporal dynamics, the overall magnitude of physiological change was quantified for each tissue parameter by calculating the absolute Δ from the beginning to the end of dialysis for each subject (Fig. 5). Among the parameters assessed, Δ water ratio was the only measure to show a statistically significant difference between groups (p<0.05 by a two-sample t-test), with subjects who experienced adverse events exhibiting notably smaller reductions or even slight increases in water ratio compared with those without adverse events. No significant group-level differences were observed for Δ $\left[{\mathrm{HbO}}_{2}\right]$, Δ [Hb], Δ [HbT], or Δ ${\mathrm{StO}}_{2}$. The distinct behavior of Δ water ratio suggests it may capture relevant fluid-handling differences associated with adverse events and warrants further investigation as a potential marker during hemodialysis. Figure S7 in Supplementary Material 1 displays the time traces of adverse events over % dialysis completion. Adverse events on average occurred at 51.72%±24.84% of dialysis completion.

### 3.3 Tissue Reduced Scattering Parameters of Patients with Adverse Versus Nonadverse Events

**Fig. 6:**
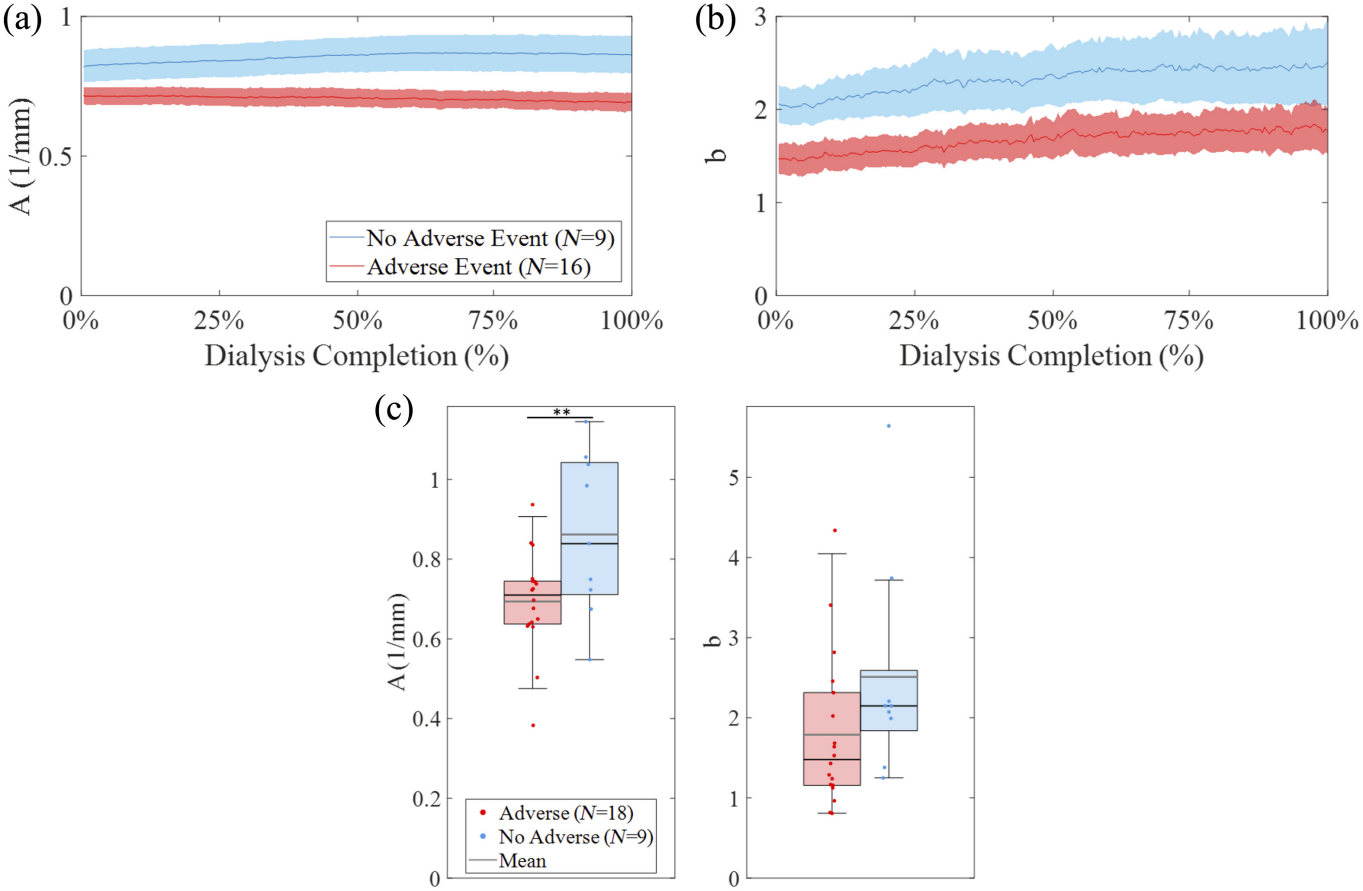
(a), (b) Longitudinal group-averaged reduced scattering amplitude and slope (A and b, respectively) over normalized dialysis time for adverse events (red, N=16) and non-adverse (blue, N=9) subjects. Two adverse subjects with short-duration sessions (t≤114  min) were excluded from these plots. (c) Box plots of absolute values of reduced scattering amplitude and slope (A and b, respectively) at the end of dialysis for subjects with adverse events (red, N=18) and nonadverse (blue, N=9). A two-sided t-test was used to assess whether group means differed significantly from zero (p<0.05). Statistically significant changes were observed in reduced scattering amplitude A. We note that the two adverse subjects excluded from panels (a) and (b) are shown in panel (c).

To complement the longitudinal analysis of chromophores and tissue water content, optical reduced scattering parameters were also examined derived from the power-law fit,^55^ namely, the reduced scattering amplitude (A) and slope (b), shown in Figs. 6(a) and 6(b). A discriminant analysis of the absolute and delta reduced scattering parameters showed that the absolute values provided better performance in predicting adverse versus nonadverse event groups (see Sec. 3.4 for details of the discriminant analysis). Subjects who did not experience adverse events exhibited higher baseline A values and increasing b over the course of dialysis, whereas those with adverse events showed comparatively flatter profiles. Comparison of reduced scattering amplitude and slope at the end of hemodialysis is displayed in Fig. 6(c). A two-sided unpaired t-test was performed to assess statistical differences between the groups. Of the parameters evaluated, at 100% dialysis completion, reduced scattering amplitude A (p=0.0130) was significantly different between groups.

### 3.4 Discriminant Analysis

**Fig. 7:**
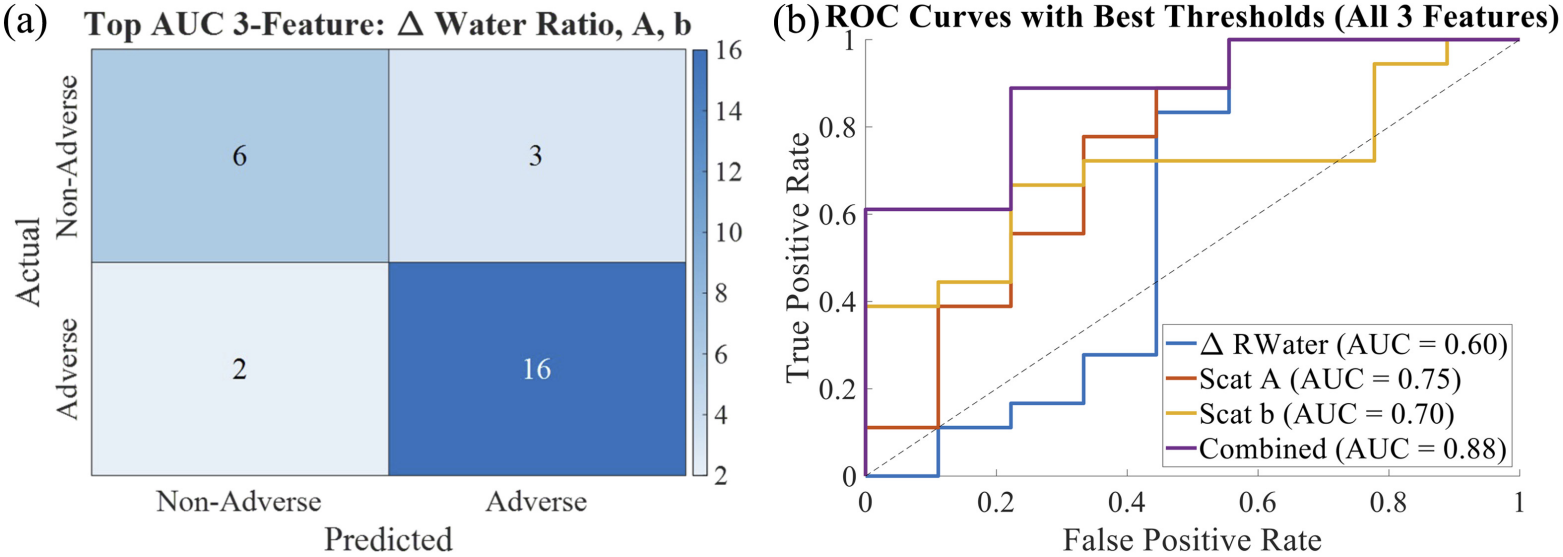
(a) Confusion matrix from a top-performing three-feature classification model (Δ water ratio, A, b). (b) ROC curves showing the classification performance of individual features and the combined model. Δ water ratio, A, and b at 100% dialysis completion individually gave low AUC values of 0.6, 0.75, and 0.7, respectively, with the combined model achieved an AUC of 0.88.

All measured optical parameters (including chromophore extractions, water ratio, and reduced scattering parameters) were included in a classification analysis to evaluate their ability to differentiate between groups. A three-feature combination of Δ water ratio, A, and b yielded the highest classification performance of all tested features, with 16 true positives, 6 true negatives, 3 false positives, and 2 false negatives [Fig. 7(a)]. Although individual features gave relatively low area under the curve (AUC) values [Fig. 7(b)], the combined model achieved an AUC of 0.88 [Fig. 7(b)] for the parameters measured at 100% dialysis completion. These results suggest that multivariate integration of reduced scattering amplitude and slope and water ratio parameters may enhance the detection of adverse events in this dataset. Notably, this feature set also demonstrated predictive value earlier in treatment, achieving an AUC of 0.92 at 25% dialysis completion. These results suggest that the FD-Bb-NIRS system may provide clinical utility in early identification of at-risk patients. Figure S6 in Supplementary Material 1 shows the AUC across the three features over all timepoints during dialysis.

### 3.5 Comparison with Crit-Line and Blood Pressure Metrics

**Fig. 8:**
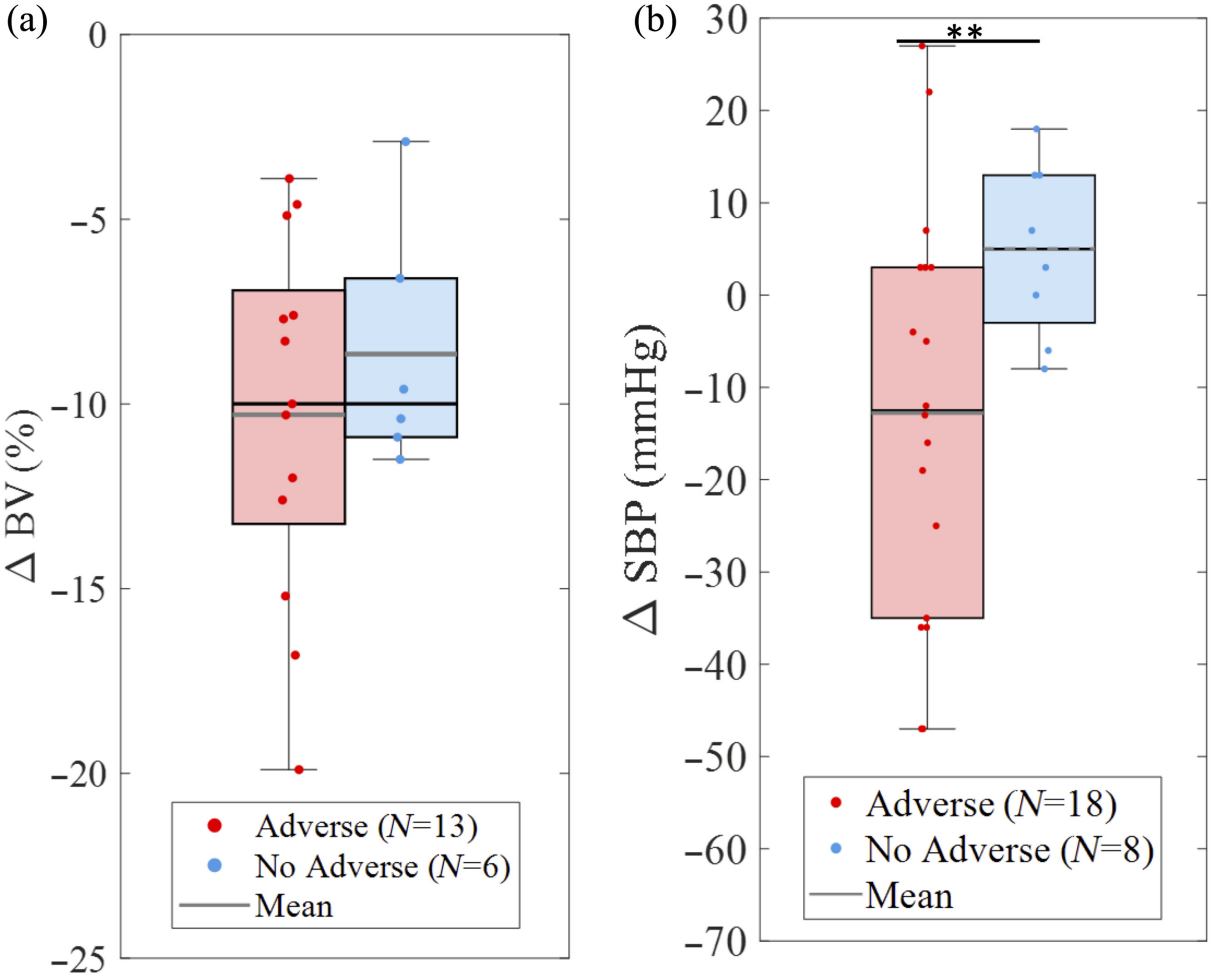
(a) Box plots of absolute change in relative blood volume (Δ BV) from start to end of dialysis, as measured by Crit-Line. Subjects with adverse events (red, N=13) and without (blue, N=6) presented similar means. No statistically significant difference was observed between groups. (b) Box plots of the absolute change in systolic blood pressure (Δ SBP) from start to end of dialysis subjects with adverse events (red, N=18) and without (blue, N=8). Blood pressure data were not available to one subject due to a dialysis machine malfunction. A two-sided t-test (p=0.039) shows that there is a statistically significant difference between groups.

To evaluate whether Crit-Line measurements of relative blood volume change (Δ BV (%)) could be used to differentiate between patient groups with adverse versus nonadverse events, percent changes in hematocrit-derived BV were compared from the start to the end of hemodialysis [Fig. 8(a)]. Although the adverse event group exhibited a wider distribution of Δ BV values, there was no statistically significant difference between groups. These findings suggest that Δ BV alone, as measured by Crit-Line, was not a reliable discriminator of adverse events within this cohort. This interpretation should consider that the sample size of Crit-Line (N=19) is smaller than the overall study population (N=27). Longitudinal trends in Crit-Line blood volume are shown in Fig. S5 in Supplementary Material 1.

A summary of all measured physiological parameters, including chromophores, Δ BV, and systolic blood pressure, is provided in Table S2 in Supplementary Material 1. Among all variables assessed, Δ water ratio was the only optical marker statistically associated with adverse events. Δ SBP was also identified as a potential statistical marker [Fig. 8(b)]; however, it is noteworthy that 16 of the 18 subjects who experienced an adverse event had at least one documented episode of hypotension during dialysis. Blood pressure data were not available for one subject due to a dialysis machine malfunction. Thus, no SBP data were collected for that subject in Fig. 8(b). Figures S8 and S9 in Supplementary Material 1 show the longitudinal and delta chromophore plots of Hypotensive versus nonhypotensive subject groups.

## 4 Discussion

We presented here the findings from a clinical study in which an FD-Bb-NIRS system was used to monitor optical and physiological changes during hemodialysis and evaluated their association with intradialytic adverse events. To date, few optical studies have systematically examined chromophore dynamics during hemodialysis, and none, to the best of our knowledge, have reported FD + CW NIRS measurements to longitudinally monitor tissue hydration status.

Among the parameters analyzed, Δ water ratio and the scattering A parameter were the only metrics to show statistically significant differences between subjects with and without adverse events. Although chromophore trends such as those seen in ${\mathrm{HbO}}_{2}$, Hb, and ${\mathrm{StO}}_{2}$ exhibited similar overall magnitudes between groups, Δ water ratio diverged substantially. The reduced scattering parameters, A and b, demonstrated clear longitudinal differences and contributed to the top-performing three-feature classification model. It is notable that Crit-Line-derived blood volume changes (Δ BV) were not able to differentiate between groups. Although Δ SBP also reached statistical significance, we note that the majority of adverse events (16 of 18) were hypotension-related.

The observed group-level divergence in Δ water ratio may reflect a mismatch between the prescribed ultrafiltration rate and the subject’s vascular refill capacity (plasma refill rate) or tissue water clearance, potentially contributing to the onset of adverse events. A declining water ratio in nonadverse event subjects is consistent with effective interstitial and intracellular water removal, whereas the relative stability or increase in the adverse event group may reflect a disparity between ultrafiltration rate and vascular refill capacity, leading to insufficient compensation and a higher risk of hypotensive events. These findings suggest that optical measurements of tissue water behavior (interstitial volume) may precede the development of dialysis-related adverse events and thus can be leveraged as an early sign of hemodynamic instability not apparent in currently available clinical measurements such as blood pressure and hematocrit.

There is limited prior literature characterizing chromophore changes in patients during hemodialysis. The observed hemodynamic (e.g., HbT increasing by ∼17  μM) was in agreement with previously published FD measurements taken in the context of dialysis by De Blasi et al., who reported a HbT increase of ∼18  μM.^32^ In addition, the absolute tissue absorption and reduced scattering values measured in the study population aligned with more broadly published ranges for skin and muscle.^55^ Other related work includes the study of Pierro et al., in which cerebral blood flow was measured in hemodialysis patients using FD-NIRS. In their preliminary application of coherent hemodynamics spectroscopy (CHS), significantly prolonged capillary and venous transit times were shown in hemodialysis patients compared with healthy controls, potentially indicating microvascular dysfunction.^56^ The work presented here differs from prior studies in physiological focus and analytical approach. Previous works have monitored changes in oxygen saturation and cerebral tissue oxygenation in hemodialysis patients using FD-NIRS and focused on extracting oxygenated and deoxygenated hemoglobin concentrations.^54^^,^^56^^,^^57^ Here, we utilized a broadband wavelength range allowing for accurate assessment of absolute hemoglobin concentrations together with water and lipid content.

Differences in optical reduced scattering are more challenging to interpret likely due to multiple contributing factors such as edema, cellular swelling, and changes in vascular dynamics.^25^^,^^43^^,^^58^ Prior literature has shown that as the hydration status of tissue changes, reduced scattering is altered.^43^^,^^58^ This effect is potentially caused by shifts in hydration status, altering cell packing density, thus altering the amplitude of $\mu_{s}^{\prime }$.

Other clinical methods for hemodialysis monitoring, such as optical hematocrit tracking, bioimpedance, and intravascular ultrasound assessment of inferior vena cava diameter, each have their own limitations related to cost, invasiveness, technical complexity, and limited sensitivity to physiological changes. Direct hematocrit monitoring methods, which involve blood draws, are labor-intensive and susceptible to operator variability.^59^^,^^60^ Indirect hematocrit monitoring, with the most common technology being Crit-Line, optically monitors *ex vivo* blood being filtered through the hemodialysis machine and measuring relative blood volume changes. Crit-Line was designed to estimate the risk of adverse events and the likelihood of achieving dry weight during a session.^59^^–^^62^ However, measurements have been shown to be inconsistent in predicting adverse events relating to fluid volume shifts, limiting their clinical reliability.^59^^–^^61^^,^^63^ Another method currently used for fluid monitoring is bioimpedance, which utilizes a small electrical current to probe core and limb composition. Bioimpedance measurements are influenced by electrolyte shifts and temperature.^60^^,^^61^^,^^64^ In intravascular ultrasound (IVUS), an intravenous ultrasound measures the diameter of the inferior vena cava to assess intravascular volume and to predict changes in volume as a response to dialysis. This method requires catheterization, which is invasive and often unavailable in routine clinical settings.^1^^,^^15^^–^^17^^,^^47^^,^^59^^,^^60^ Current approaches to volume assessment during dialysis remain largely out of reach and inaccurate in current clinical settings.

In contrast to these other techniques, our optical approach offers a noninvasive and physiologically specific alternative, with real-time sensitivity to changes in both chromophore and water content. All of the above-discussed clinical technologies focus on intravascular volume assessment, whereas >60% of total body water remains in extravascular spaces, for which there are no clinical methods to assess its flux.^65^

A notable limiting factor in this study is that the participant cohort consisted of inpatient dialysis patients, who may differ clinically from the broader, stable, outpatient population, potentially limiting generalizability. Common comorbidities among study participants included: congestive heart failure, diabetes, hypertension, chronic infections, and anemia.^2^^,^^7^^,^^59^ On average, 1.6±0.8  L of fluid were removed during dialysis. This volume is lower than typically observed in outpatient dialysis populations, which may reflect the higher comorbidity burden of the inpatient study cohort. Previous literature reported an accepted removal rate of 10 to 13  mL/h/kg over an ∼3.75-h outpatient dialysis session. Thus, for an individual attending outpatient dialysis with a weight of 70 kg, their expected ultrafiltration goal would be between 2.6 to 3.4 L.^2^^,^^11^^,^^66^ The median dialysis duration was 3 h (range: 1 to 4 h); however, nine sessions were terminated early due to the onset of severe intradialytic complications such as the possibility of stroke, severe leg cramping, low blood pressure, blood clots, or onset of malaise or anxiety. These clinical constraints further contributed to variability in fluid removal and treatment duration.

Although Δ water ratio demonstrated statistically significant group differences, the small sample size (N=27) is a limitation of this study. However, our objective was to examine the feasibility of the FD-Bb-NIRS system as a preliminary clinical study. Future studies with a higher power, larger, more diverse cohorts, and a broader range of adverse event types will be important to validate the discriminatory power of the water ratio and other optical metrics. In addition, a limitation of this study was the use of a homogeneous inverse model, which does not account for the layered structure of tissue (skin, adipose, and muscle).^67^^,^^68^ This simplification may lead to inaccuracies in absolute estimates of chromophore extractions and may have implications related to the accurate extraction of water and lipids. In the future, this might be improved with multilayer Monte Carlo models to account for the heterogeneous structure of tissue. Another limitation is related to splitting the FD source illumination to more than one tissue location. This allowed for more optical power to be distributed across a larger skin area, thus enabling overall higher illumination optical power while staying within American Standard Safety Institute limits. Although this strategy has previously been used by our group and others,^47^^,^^69^^,^^70^ it assumes tissue homogeneity. Finally, we note that improved quantification of water and lipids may be achievable by utilizing short-wave infrared measurements, as we have recently demonstrated.^43^

Finally, although the current study focused on hemodialysis, the measurement of tissue water dynamics may have broader clinical relevance. Similar methodologies could be used for assessing edema in patients with cardiovascular disease.^50^^,^^71^^,^^72^ FD-Bb measurement techniques may also be useful for noninvasive quantification of hydration level and body composition,^73^ such as monitoring intentional weight gain or loss, or in sports medicine.^74^

## 5 Conclusion

We conducted a pilot clinical study using a combined FD-Bb-NIRS system to monitor tissue optical changes during hemodialysis. Among the parameters evaluated, Δ water ratio and the scattering A parameter emerged as the only metrics to show statistically significant differences between subjects with and without adverse events. Changes in water and reduced scattering parameters contributed to multivariate classification. These findings support further investigation of FD-Bb-NIRS in a larger and more diverse cohort as a tool to guide ultrafiltration in hemodialysis, a predictive tool for impending adverse events, and most importantly, to assist in objectively defining the dry weight on hemodialysis.

## Supplementary Material

10.1117/1.BIOS.3.1.015003.s01

10.1117/1.BIOS.3.1.015003.s02

## Data Availability

Data points contained in the figures of this article are available in Supplementary Material 2.
