# Supplementary material for: Frequency-domain broadband near-infrared spectroscopy for noninvasive monitoring of fluid volume status during hemodialysis
Source: Biophotonics Discov. 2026 Feb 4;3(1):015003. doi: 10.1117/1.BIOS.3.1.015003 (PMC13097106; doi:10.1117/1.BIOS.3.1.015003)
Supplement: Supplementary file 1 [file BIOS_003_015003_SD001.pdf]

Supplemental Figures

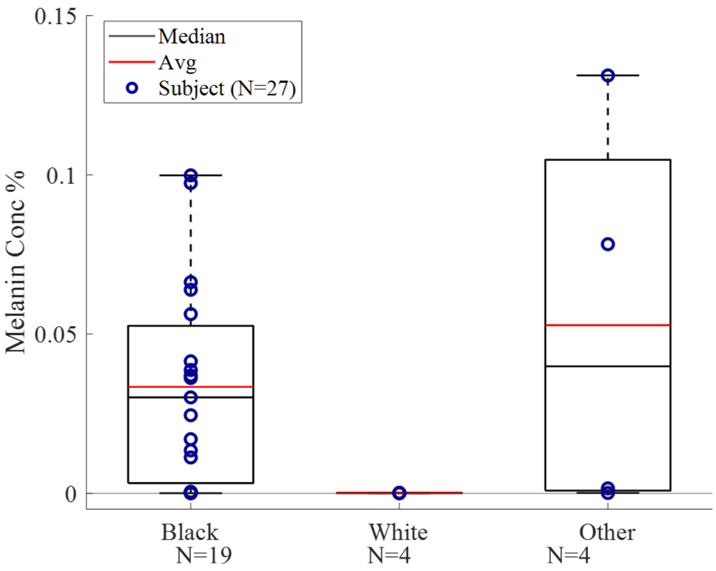

Fig S1 Box plot of [Melanin] across subject's (N=27) self-reported race, stratified by self-identified race into three groups: Black (N=19), White (N=4), and Other (N=4). [Melanin] was derived from baseline measurements taken of pre-dialysis. These findings reflect expected physiological variability in epidermal melanin content across racial groups

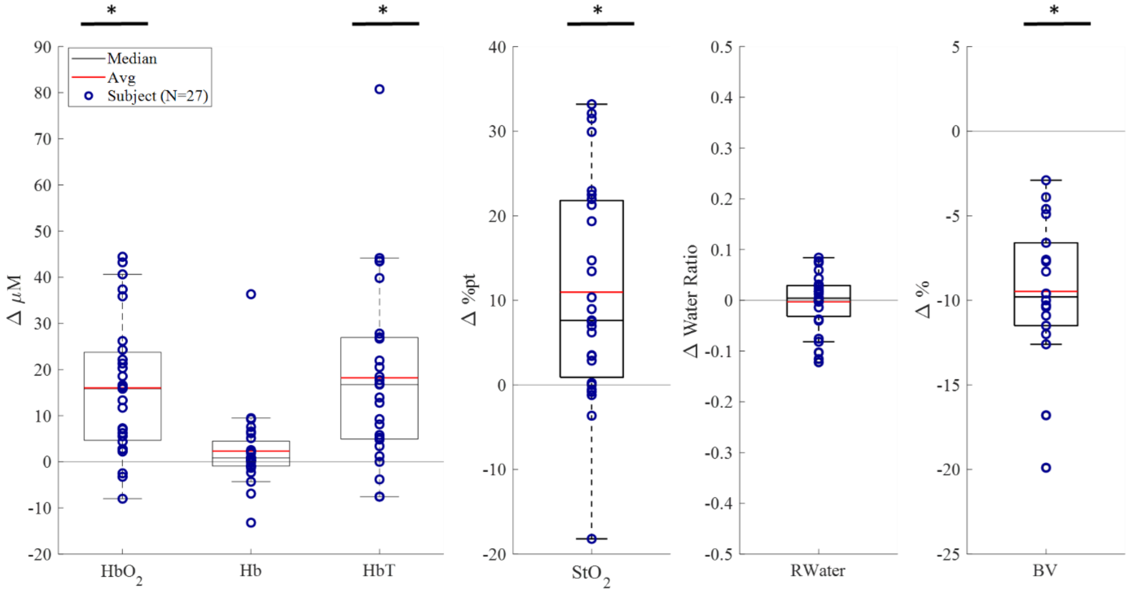

Fig S2 Box plots represent the change (Δ) in the measured chromophore derived parameters for each subject (N=27). oxyhemoglobin (HbO_2), deoxyhemoglobin (Hb), total hemoglobin (HbT), tissue oxygen saturation (StO_2), Water Ratio (RWater), and relative blood volume (BV) derived from crit line (N=19). A one-sample t-test was used to assess whether group means differed significantly from zero ($p < 0.05$). Statistically significant changes were observed in HbO_2, HbT, StO_2, and BV across the cohort.

	Adverse Events		No Adverse Events	
**Total**	N=18		N=9	
**Crit-Line**	N=13		N=6	
**Sex**				
Male	N=11		N=5	
Female	N=7		N=4	
**Race**				
Black	N=12		N=7	
White	N=1		N=2	
Other	N=4		N=0	
**Ethnicity**				
Hispanic	N=6		N=1	
Non-Hispanic	N=12		N=7	
Other	N=0		N=1	
	**Mean ± Stdev**		**Mean ± Stdev**	
**Age**	59.7 ± 14.8 (Y.O)		57.0 ± 13.7 (Y.O)	
**Height**	1.72 ± 0.1 (M)		1.68 ± 0.1 (M)	
**Weight**	78.8 ± 16 (Kg)		79.0 ± 15.2 (Kg)	
**Fluid Removed**	1.5 ± 0.9 (L)		1.7 ± 0.5 (L)	
	**Median**	**Range**	**Median**	**Range**
**Dialysis duration**	3	1-3.5 (Hr)	3	2.25-3.5 (Hr)

Table S1 Demographic and treatment characteristics of subjects stratified by occurrence of intradialytic adverse events (N=18 with adverse events, N=9 without). Values are presented as mean ± standard deviation unless otherwise indicated. Fluid removed represents the average ultrafiltration volume per session. Crit-Line data was available for a subset of participants (N=13 with adverse events, N=6 without) and refers to subjects whose hematocrit was continuously monitored during dialysis via the Fresenius Crit-Line IV Monitor.

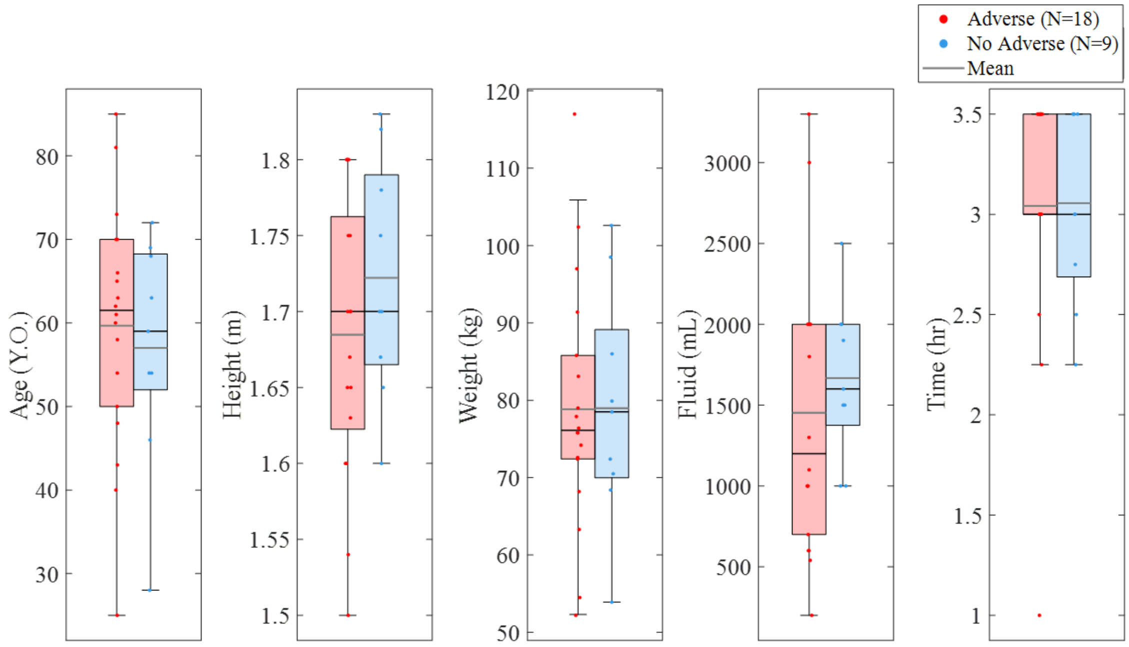

Fig S3 Box plots of the demographic and treatment characteristics are shown for subjects who experienced intradialytic adverse events (red, N=18) and those who did not (blue, N=9). Parameters include age, height, weight, fluid volume removed, and dialysis session duration. There is no statistically significant difference in the characteristics between the two groups.

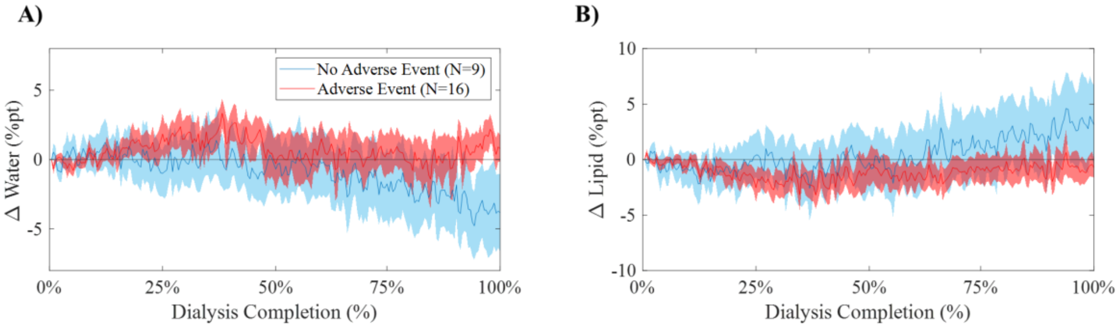

Fig S4 Longitudinal changes of **A)** Δ[Water] (%) and **B)** Δ[Lipid] (%), across subjects with (red, N=16) and without (blue, N=9) adverse events during dialysis. Solid lines represent mean absolute chromophore concentration change; shaded regions represent the standard error. Subjects who experienced adverse events demonstrated a relative increase in [Water] during dialysis, while those without adverse events showed progressive decreases in [Water].

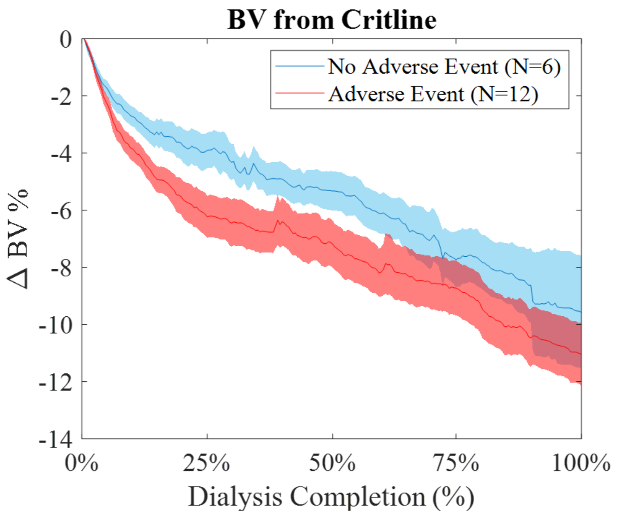

Fig S5 Longitudinal changes in relative blood volume change (ΔBV%) measured by Crit-Line over the course of dialysis for subjects with (N = 12, red) and without (N = 6, blue) intradialytic adverse events. Solid lines represent mean BV change; shaded regions represent the standard error. One subject was excluded from the analysis due to total dialysis time $\leq$ 114 min as described in Section 3.2. ΔBV% trends between groups were not statistically different.

	Adverse Events		No Adverse Events		
**Δ**	**Subjects**	**Mean ± Stdev**	**Subjects**	**Mean ± Stdev**	**P-Value (2-Sided t-test)**
**$[HbO_2]$ (μM)**	N=18	14.52 ± 15.82	N=9	15.96 ± 12.21	0.814
**[Hb] (μM)**	N=18	2.25 ± 6.49	N=9	1.57 ± 3.88	0.776
**[HbT] (μM)**	N=18	16.78 ± 17.64	N=9	17.53 ± 14.43	0.912
**StO_2 (% pt)**	N=18	10.62 ± 12.41	N=9	10.32 ± 15.78	0.956
**Water Ratio**	N=18	0.012 ± 0.035	N=9	-0.037 ± 0.085	**0.033**
**SBP (mmHg)**	N=18	-12.77 ± 21.94	N=8	5.00 ± 9.41	**0.039**
**BV (%, Crit-Line)**	N=13	-10.29 ± 4.89	N=6	-8.65 ± 3.30	0.468

Table S2 Comparison of absolute changes (Δ) in optical and clinical parameters between subjects with (N=18) and without (N=9) intradialytic adverse events. Reported values reflect the mean ± standard deviation of changes from baseline to end of dialysis treatment for optically measured biomarkers: oxyhemoglobin ($[HbO_2]$), deoxyhemoglobin ([Hb]), total hemoglobin ([HbT]), tissue oxygen saturation (StO_2), and water ratio. Clinical measurements including relative blood volume (BV) as measured by Crit-Line, and systolic blood pressure (SBP). A two-sided unpaired t-test was performed to assess statistical differences between groups. Of the parameters evaluated, Water Ratio ($p = 0.0331$) and SBP ($p = 0.039$) were significantly different between groups, with adverse event subjects exhibiting a relative increase in water ratio and a more pronounced decline in systolic blood pressure.

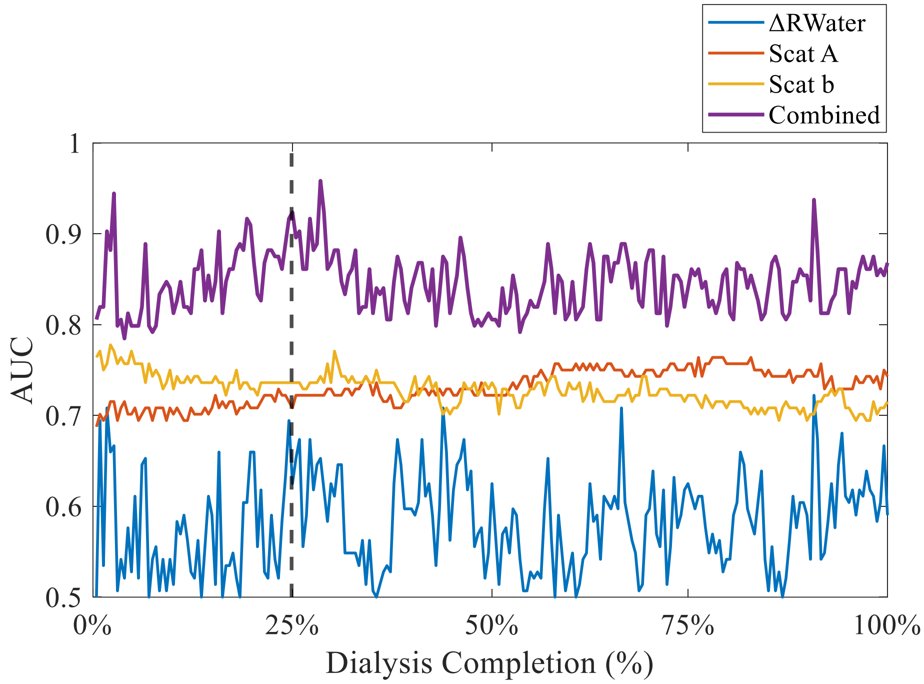

Fig S6 The AUC from the top-performing 3-feature classification models (Δ Water Ratio, A, b) and the combined features over the normalized time course of dialysis completion.

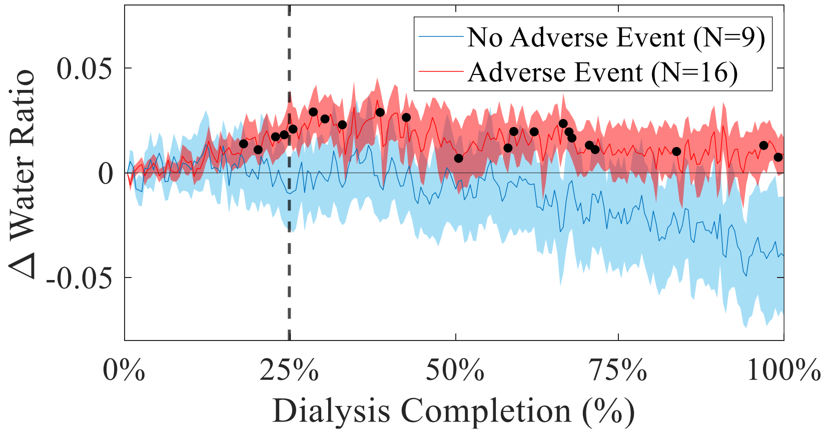

Fig S7 Time-normalized traces (mean ± standard error) comparing chromophore extractions of subjects with adverse events (red, N = 16) and without (blue, N = 9) of the Δ Water Ratio (Two subjects with short-duration sessions ($t \leq 114$ min) were excluded from the normalized time trace). The black dots overlay indicate the timestamp of adverse events occurring. The average adverse event occurred at 51.72% +/- 24.84 % of dialysis completion

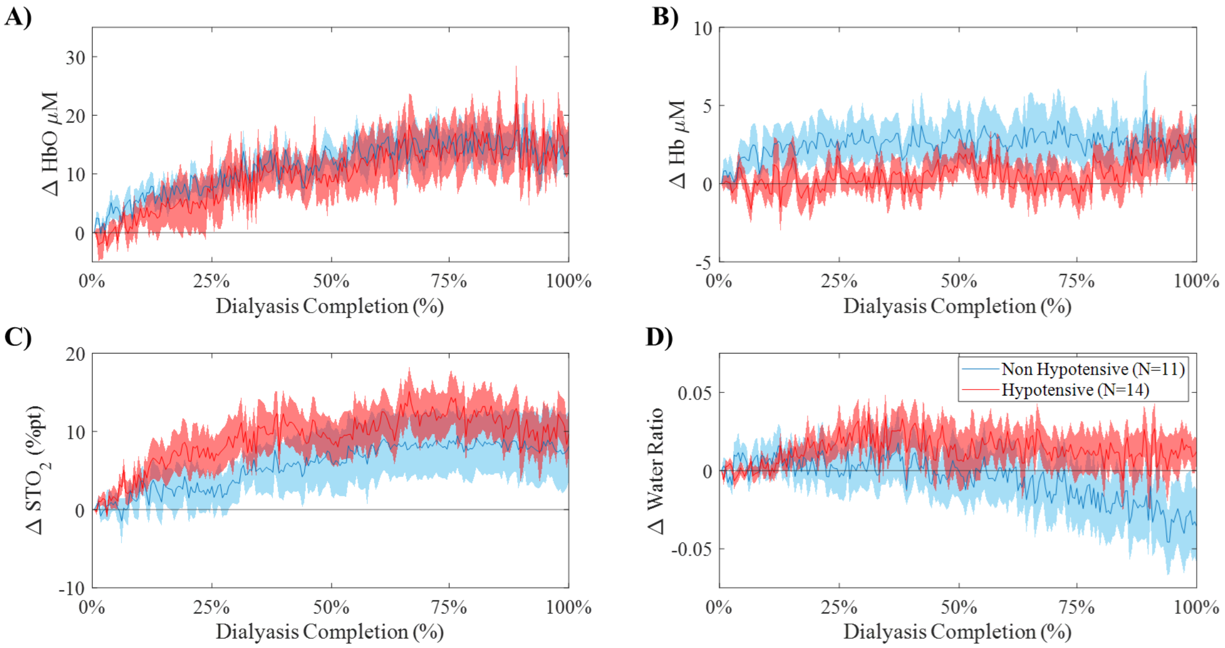

Fig S8 Time-normalized traces (mean ± standard error) comparing chromophore extractions of subjects with Hypotensive events (red, $N = 14$) and without (blue, $N = 11$) **A)** Δ [HbO_2] **B)** Δ [Hb] **C)** Δ StO_2 **D)** Δ Water Ratio. Two subjects with short-duration sessions ($t \leq 114$ min) were excluded.

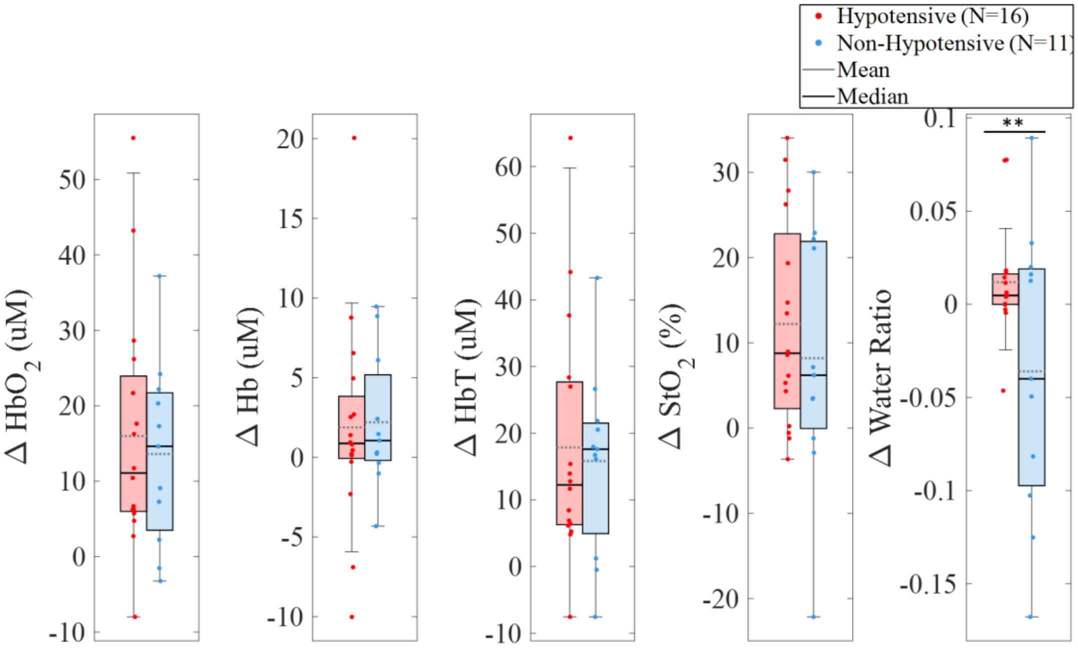

Fig S9 Boxplots of subject values and group means for the absolute Δ [HbO_2], Δ [Hb], Δ [HbT], Δ StO_2, and Δ Water Ratio from the beginning to end of dialysis. Hypotensive subjects are shown in red ($N = 16$), and non-Hypotensive subjects in blue ($N = 11$). A significant difference was observed for Δ Water Ratio ($p < 0.05$). No other parameters reached statistical significance.
